# Brain Metabolomics Reveal the Antipyretic Effects of Jinxin Oral Liquid in Young Rats by Using Gas Chromatography–Mass Spectrometry

**DOI:** 10.3390/metabo9010006

**Published:** 2019-01-01

**Authors:** Wenjuan Qian, Jinjun Shan, Cunsi Shen, Rui Yang, Tong Xie, Liuqing Di

**Affiliations:** 1 Jiangsu Key Labortory of Pediatric Respiratory Disease, Institute of Pediatrics, Nanjing University of Chinese Medicine, Nanjing 210023, China; 20161325@njucm.edu.cn (W.Q.); cunsishen@126.com (C.S.); 20171349@njucm.edu.cn (R.Y.); sunnyxyl1021@163.com (T.X.); 2 Medical Metabolomics Center, Nanjing University of Chinese Medicine, Nanjing 210023, China; 3 Jiangsu Engineering Research Center for Efficient Delivery System of TCM, School of Pharmacy, Nanjing University of Chinese Medicine, Nanjing 210023, China

**Keywords:** metabolomics, pyrexia, gas chromatography-mass spectrometry, Jinxin oral liquid, biomarkers

## Abstract

Pyrexia is considered as a part of host’s defense response to the invasion of microorganisms or inanimate matter recognized as pathogenic or alien, which frequently occurs in children. Jinxin oral liquid (JXOL) is a traditional Chinese medicine formula that has been widely used to treat febrile children in China. Experimental fever was induced by injecting yeast into young male Sprague-Dawley rats (80 ± 20 g) and the rectal temperature subsequently changed. Four hours later, the excessive production of interleukin (IL)-1β and prostaglandin (PG) E2 induced by yeast was regulated to normal by JXOL administration. A rat brain metabolomics investigation of pyrexia of yeast and antipyretic effect of JXOL was performed using gas chromatography-mass spectrometry (GC-MS). Clear separation was achieved between the model and normal group. Twenty-two significantly altered metabolites were found in pyretic rats as potential biomarkers of fever. Twelve metabolites, significantly adjusted by JXOL to help relieve pyrexia, were selected out as biomarkers of antipyretic mechanism of JXOL, which were involved in glycolysis, purine metabolism, tryptophan mechanism, etc. In conclusion, the brain metabolomics revealed potential biomarkers in the JXOL antipyretic process and the associated pathways, which may aid in advanced understanding of fever and therapeutic mechanism of JXOL.

## 1. Introduction

There are a wide range of diseases that can cause pyrexia. Pyrexia is a kind of defensive response when pathogens and microorganisms invade the host. Meanwhile, fever is one of the main reasons children and infants come for medical evaluation and often leads to administration of antipyretics [1].

Ibuprofen (IB), acetaminophen, and aspirin are commonly used antipyretics for reducing high body temperature quickly. IB is one of the most widely used western medicine for antipyretic, analgesic, and anti-inflammation. In China, traditional Chinese medicine (TCM) usage is regular policy because of the long-time effect and low toxicity [2]. TCM has been used to treat pediatric fever by practitioners for many years [3,4]. Jinxin oral liquid (JXOL) is a traditional Chinese medicine formula modified from an ancient formula ma-xing-shi-gan-tang and consists of eight herbal or mineral medicine, which are *Ephedra sinica Stapf.*, *Prunus armeniaca L.*, *Morus alba L.*, *Scutellariae baicalensis Georgi.*, *Peucedanum praeruptorum Dunn.*, *Lepidium apetalum Willd.*, *Polygonum cuspidatum Sieb.* and *Gypsum (CaSO_4_·2H_2_O).* JXOL has been patented in China [5], whose eight main bioactive compounds have been detected and analyzed by ultra-performance liquid chromatography [6]. Clinically, JXOL has been widely used in treatment of pediatric respiratory diseases, especially respiratory virus infection and hyperthermia accompanied [7,8,9,10]. However, the underlying mechanism of its antipyretic effect was unclear.

Metabolomics is the untargeted and targeted analysis of small molecular metabolites, which has been widely applied in revealing new biomarkers of disease and finding out the underlying mechanisms of clinical drugs [11]. Mass spectrometry (MS) coupled with chromatographic separation apparatus, such as gas chromatography (GC), has been used as a powerful tool to analyze complex biological samples [12,13]. GC-MS has the ability to detect the chromatographic peaks sensitively, accurately and reliably [14]. Combined with National Institute of Standards and Technology database (NIST), the Human Metabolome Database (HMDB), Kyoto Encyclopedia of Genes and Genomes (KEGG), GC-MS metabolomics has great potency in toxicology, disease diagnosis, biomarker discovery [15], and pharmaceutical fields [16,17,18].

Previous metabolic researches on fever usually focused on easily accessible bio-fluid samples to explore metabolic changes in brain, such as serum, plasma, and urine [2,3,4,19,20]. However, due to blood-brain-barrier (BBB), which can tightly control the passage of molecules between brain and blood, the extent that biomarkers from bio-fluid samples could reflect the brain metabolism is not clear [21]. Therefore, brain metabolomics has been used in our investigation to monitor the antipyretic effect of JXOL. 

As we all know, many differences exist between young brain and adult brain that can be presented in energy metabolism pathways [22], lipids metabolism [23], and so on. Adult rats based brain metabolomics was not appropriate to study pediatric disease and drugs. Thus, we used young rats rather than adult rats to investigate the mechanisms of pyrexia in children and JXOL.

In this study, we collected brain tissue samples from young rats and performed untargeted metabolomics research on a GC-MS platform to explore biomarkers of yeast-induced hyperthermia in rat brain. Afterwards, we found out 12 JXOL therapy related metabolites that may have participated in regulating the state of brain microenvironment, anti-inflammatory, and antipyretic pathways in brain. These results provide more evidence to better understanding the underlying antipyretic mechanism of JXOL.

## 2. Results

### 2.1. Antipyretic and Anti-Inflammation Effects of IB and JXOL

**Figure 1:**
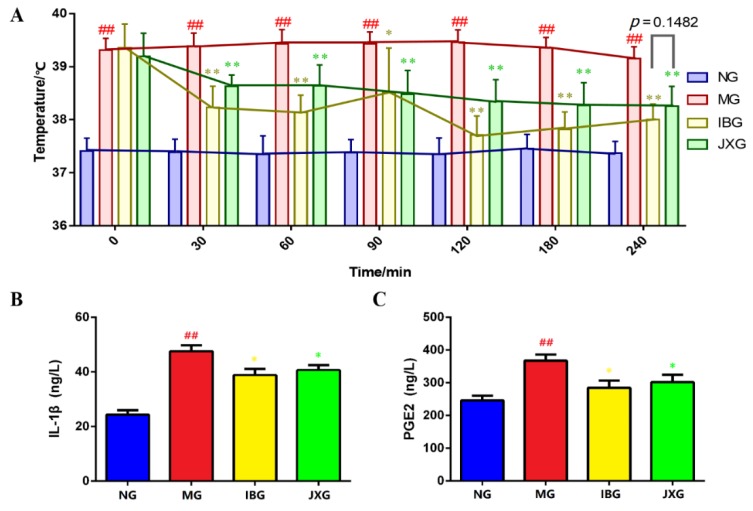
Antipyretic and anti-inflammation effects of IB and JXOL. A: The rectal temperatures were measured at 0, 30, 60, 90, 120, 180, and 240 min after drug administration. B: Effects of JXOL on the production of IL-1β and PGE2. (# *p* < 0.05, ## *p* < 0.01, vs. NG; * *p* < 0.05, ** *p* < 0.01, vs. MG).

The rectal temperatures of rats in each group before and after drugs administration were recorded to monitor the body temperature changes. Rectal temperatures were measured at 0, 30, 60, 80, 90, 120, 180, and 240 min after drug administration. As shown in Figure 1A, the temperature fluctuations of normal group (NG) changed slightly. At the time-point of 0 min, the rectal temperatures of rats which were injected with yeast, in other words, the temperatures of model group (MG), ibuprofen group (IBG), and Jinxin group (JXG) were significantly higher than the temperature of NG (*p* < 0.05), indicating that the yeast-induced pyretic model was successful. After drug administration, the rectal temperatures of IBG and JXG decreased quickly and last about 240 min. In addition, the body temperature of IBG continued to drop for 120 min and then began to rise, while the temperature of JXG kept a downward trend for four hours and finally maintain similar antipyretic effect compared with IBG (*p* = 0.1482). 

Fever causes excessive inflammation in the brain [24]. IL-1β is a kind of pro-inflammatory mediators, which plays an important role in the development of excessive inflammation caused by fever. In plasma, the concentration of IL-1β of MG increased significantly compared to NG (*p* < 0.01) (Figure 1B). In the meantime, the level of IL-1β of IBG and JXG decreased significantly compared to MG (*p* < 0.05). PGE2 production increased obviously because of yeast injection. After drug administration, the level of PGE2 of IBG and JXG turned back to a normal level and show statistical significance compared to MG.

### 2.2. Brain Metabolomics Profile and Multivariate Data Analysis

**Figure 2:**
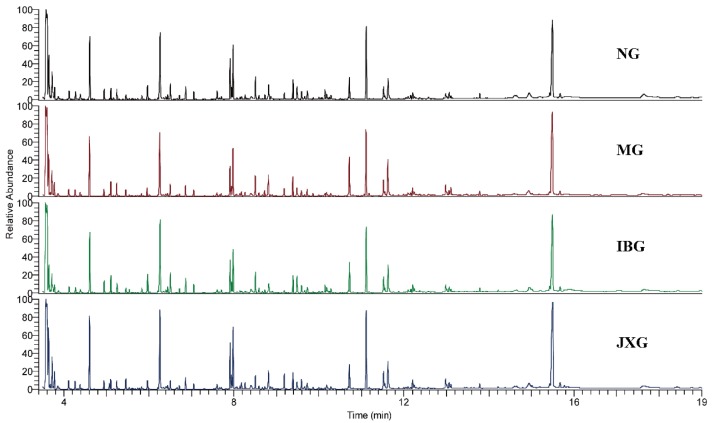
Typical GC-MS total ion chromatograms (TIC) of brain samples from NG, MG, IBG, and JXG.

Low-molecular-weight metabolites were represented as chromatographic peaks. Typical GC-MS total ion chromatograms (TIC) of brain samples from NG, MG, IBG, and JXG were shown in Figure 2. A total of 112 metabolites whose peak height intensity were the basis of subsequent statistical analysis were accurately identified, including amino acids, carbohydrate, glycerides, unsaturated fatty acids, pyrimidines, purines, and so on.

**Figure 3:**
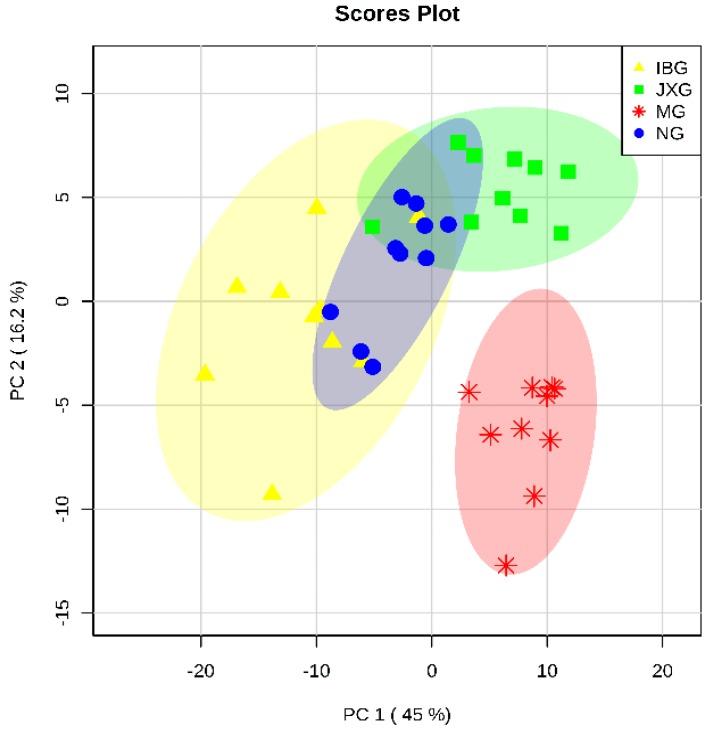
PCA scores scatter plot of brain metabolites.

In this study, a multivariate analysis method of Principal Component Analysis (PCA) was performed to visualize the similarities and differences among four groups. The PCA scores scatter plot was shown in Figure 3. As we can see from Figure 3, a good separation was demonstrated among MG and NG, IBG, JXG, which indicated that yeast-induced fever significantly altered the levels of endogenous metabolites in rat brain. Besides, the separation among NG, IBG, and JXG was not so good, which suggested that the perturbed metabolites in rat brain might be regulated to a normal level by applying IB or JXOL.

### 2.3. Identification of Potential Biomarkers and the Changing Trends Among NG, MG, IBG, and JXG

**Figure 4:**
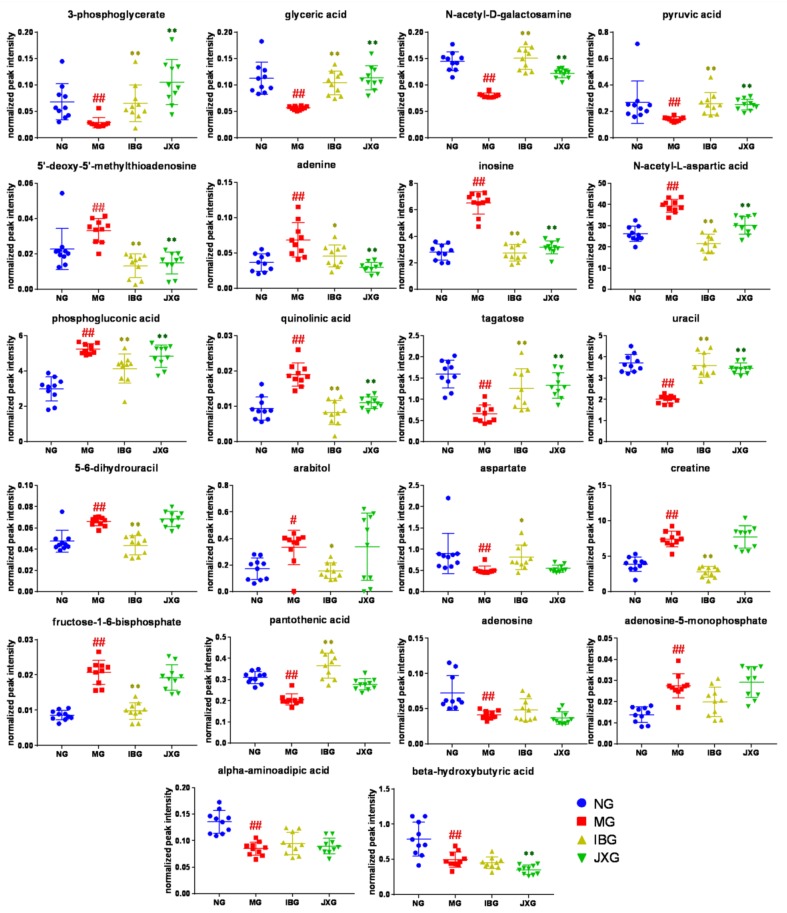
Scatter plots of significantly changed metabolites’ normalized peak intensity in rat brain samples. The x-axis shows the specific metabolite’s normalized peak intensity, and each scatter represents a rat brain sample. 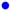 represents brain samples of NG, 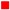 represents brain samples of MG, 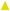 represents brain samples of IBG, and 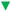 represents brain samples of JXG. The scatter plots show the mean and SD of the metabolites. # *p* < 0.05, ## *p* < 0.01, vs. NG; * *p* < 0.05, ** *p* < 0.01, vs. MG.

To reveal the antipyretic mechanism of JXOL, we chose the potential biomarkers based on two rules: For one rule, fold change (FC) value between NG and MG must be higher than 1.5; for the other rule, the *p* value between NG and MG must smaller than 0.05. After completing this step of data processing, 22 metabolites which satisfied both the criteria were selected as potential biomarkers to characterize the fever model. Detailed changing trends of these 22 metabolites among NG, MG, IBG, and JXG were shown in Figure 4. Specifically, 11 of 22 metabolites were elevated after yeast injection, containing 5′-deoxy-5′-methylthioadenosine, 5-6-dihydrouracil, adenine, adenosine-5-monophosphate, arabitol, creatine, fructose-1-6-bisphosphate, inosine, *N*-acetyl-l-aspartic acid, phosphogluconic acid and quinolinic acid. Other metabolites were downregulated, namely, 3-phosphoglycerate, adenine, alpha-aminoadipic acid, aspartate, beta-hydroxybutyric acid, glyceric acid, *N*-acetyl-d-galactosamine, pyruvic acid, pantothenic acid, tagatose, and uracil.

Additionally, JXOL administration significantly regulated 13 of 22 metabolites in rat brain. It is worth mentioning that 12 of these 13 metabolites adjusted to normal level except beta-hydroxybutyric acid in JXG, indicating that 12 metabolites mentioned above may play key roles in antipyretic effect.

## 3. Discussion

Yeast can induce local and systemic inflammatory response and then promote the secretion of inflammatory cytokines such as IL-1β. Subsequently, inflammatory signals were send to brain which cause PGE2 production and pyrexia [24]. In this study, we first monitored the rectal temperature of pyretic rats and the result indicated that JXOL has similar antipyretic effect compared with IBG in 240 min. Moreover, the production of IL-1β and PGE2 of fever rats significantly down regulated after JXOL administration (Figure 1), indicating that JXOL can reduce abnormally elevated body temperature via inhibition of inflammation.

Yeast caused excessive inflammatory response and metabolic disturbs in young rats. JXOL also can help decrease the body temperature by regulating brain metabolism. We investigated the brain metabolites profiling and potential biomarkers of yeast-induced fever and explored the underlying antipyretic mechanism of JXOL at the metabolomics level based on GC-MS. Finally, we selected 12 significantly altered metabolites as antipyretic biomarkers of JXOL in brain, in other words, JXOL may play anti-fever effect by adjusting the production of 3-phosphoglycerate, 5′-deoxy-5′-methylthioadenosine, adenine, beta-hydroxybutyric acid, glyceric acid, inosine, *N*-acetyl-d-galactosamine, *N*-acetyl-l-aspartic acid, pantothenic acid, pyruvic acid, quinolinic acid, tagatose, and uracil (Table 1 and Figure 4).

**Figure 5:**
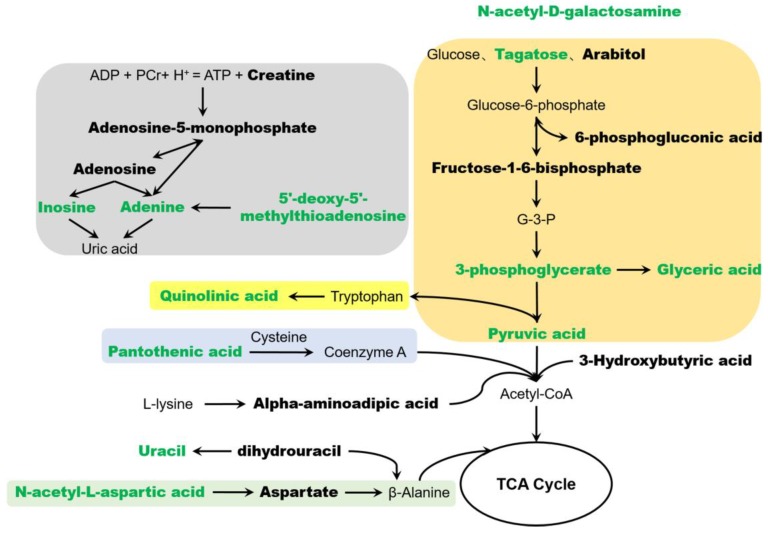
The pathways closely associated with the antipyretic and anti-inflammation effects. Bold metabolites were potential biomarkers of yeast-induced fever; green bold metabolites were antipyretic biomarkers of JXOL.

According to the distribution of metabolites in the metabolic pathways in rats, we generated a pathway network of antipyretic effect of JXOL (Figure 5). The detailed description is as follows.

Purine metabolism is critical for brain function since purines can act as metabolic signals, brain function regulator, energy transducers, endothelium-derived vasoactive nucleotides, etc. [25,26]. In this study, we found that a panel of metabolites of purine metabolism, such as adenosine-5-monophosphate, adenosine, inosine, adenine, 5′-deoxy-5′-methylthioadenosine, was significantly disturbed in pyretic young rats. While JXOL could not regulate the metabolic level of all these metabolites, as mentioned before to normal, it could adjust three of them, namely inosine, adenine, and 5′-deoxy-5′-methylthioadenosine, making the subsequent metabolism normal.

Glycolysis is a widespread phenomenon and is the basis of diverse brain activities. It is reported that more energy is required to maintain pyrexia [27], which has been proved by this experiment. From Figure 4 and Figure 5, we can easily find an increase in glycolysis in yeast-induced pyretic rat, which causes a reduced content in metabolites. However, JXOL administration regulated metabolic levels of key metabolites such as 3-phosphoglycerate, pyruvic acid, etc.

Quinolinic acid (QA) is a potential neural toxin which can be produced by kynurenine pathway of tryptophan degradation. High concentration of QA can increase glutamate release and the influx of calcium ions which will lead to excitotoxicity [28]. Besides, QA also induces mitochondrial dysfunction and inflammation [29]. In this study, the level of QA upregulated when the rats were injected with yeast, which may lead to neural injury. However, the concentration of QA dropped down after taking JXOL.

Pantothenic acid (PA), also known as vitamin B5, is a water-soluble vitamin and plays a pivotal role in health and brain function. PA metabolism is closely related to neurological function [30], and can protect biofilm system by preventing lipid peroxidation via increased GSH, which then protect cell structure from injury in brain [31]. JXOL may promote the production of PA thereby maintain brain healthy when it comes to fever.

Acetylgalactosamine and *N*-Acetyl-l-aspartic acid are widely distributed in the brain, which play important role in neurological health. Acetylgalactosamine is an important component of brain heteropolysaccharides (glycoproteins) and participates in intercellular communication. *N*-Acetyl-l-aspartic acid is a neuronal osmolyte involved in fluid balance in the brain. The results showed that disordered state induced by fever can be corrected by JXOL administration.

## 4. Materials and Methods

### 4.1. Chemicals and Reagents

Jinxin oral liquid (JXOL) was provided by Jiangsu Provincial Hospital of Traditional Chinese Medicine (Nanjing, China). The quality of JXOL was strictly controlled [6]. Baker’s yeast (Saccharomyces cerevisiae) was purchased from ANGEL YEAST CO., LTD. (no. CY0175, China).

Methoxyamine hydrochloride, pyridine, N, O-Bis (trimethylsilyl) trifluoroacetamide (BSTFA) with 1% trimethylchlorosilane (TMCS) and 1,2-^13^C-myristic acid were purchased from Sigma-Aldrich (St. Louis, MO, USA). N-hexane was purchased from ROE Scientific (St. Louis, MO, USA). Methanol of MS grade was supplied by Merck Millipore (Billerica, MA, USA). Ultra high-purity water was prepared by Millipore-Q system (Millipore Corporation, Billerica, MA, USA).

### 4.2. Animals and Model Construction

Male Sprague-Dawley rats (80 ± 20 g) were obtained from Shanghai JiesiJie Experimental Animal Co., Ltd. (Shanghai, China). All rats were acclimated for 7 days in a controlled room with temperature (23 ± 2 °C), humidity (60 ± 5%), and a light/dark cycle of 12 h before the formal study. The rats received a standard diet and water ad libitum. During the last three days, the rectal temperatures were measured twice per day using a digital thermometer for the regular rhythm of body temperature, and rats whose body temperature fluctuations were not higher than 0.3 °C were selected for the following study.

A total of 40 rats were randomly divided into four groups: normal group (NG, no injection, *n* = 10, 10 mL/kg saline i.g.); model group (MG, subcutaneous injection of yeast, *n* = 10, 10 mL/kg saline i.g.); Ibuprofen group (IBG, subcutaneous injection of yeast, *n* = 10, 50 mg/kg ibuprofen i.g.); Jinxin oral liquid group (JXG, subcutaneous injection of yeast, *n* = 10, 7.02 g/kg Jinxin oral liquid i.g.). The rats of MG, IBG, and JXG were subcutaneously injected with 20% aqueous suspension of yeast (15 mL/kg) in their back. The rectal temperatures were measured for 4 h after the yeast injection with a digital thermometer.

The animal experiments were performed under the guidelines of Animal Ethics Committee of Nanjing University of Chinese Medicine ACU-18(20151127), and the experiments were approved by the Animal Ethics Committee of our university.

### 4.3. Sample Collection and Cytokines

After the last rectal temperature measurement, all rats were anesthetized by peritoneal injection of chloral hydrate. The abdominal aorta blood was immediately collected in heparinized tubes. The obtained plasma then was frozen at −80 °C. The rat brains were also stored at −80 °C. For measuring cytokine levels in plasma, IL-1β and PGE2 ELISA kits were used by following the manufacturer’s instruction.

### 4.4. Sample Preparation and GC-MS Analysis

#### 4.4.1. Tissue Extraction

Sample preparation for GC-MS analysis was processed with improvement according to our previously published work [17,18]. Each 200 mg of frozen brain tissue (whole brain) were homogenized in 4 mL MeOH. A 600 μL of homogenate was added with 20 μL of MeOH containing IS (1,2-^13^C2-Myristic acid) which final concentration was 10 μg/mL and vortexing for 3 min. The mixture was then centrifuged at 14,000 rpm for 10 min and 300 μL of supernatant was transferred into a new tube. The supernatant was then dried at 45 °C and 15 kPa to allow the evaporation of MeOH. After drying, samples were reconstituted in 50 μL of 15 mg/mL methoxyamine pyridine and vortexed for 5 min before oscillating for 1.5 h at a speed of 300 rpm in a thermostatic oscillator at 30 °C. Then, 50 μL of N, O-Bis (trimethylsilyl) trifluoroacetamide (BSTFA) were added and shaken for another 0.5 h at 37 °C. Finally, after derivation, the mixture was centrifuged at 18,000 rpm for 10 min before injecting into GC-MS.

#### 4.4.2. GC-MS Conditions

GC-MS analysis was performed using Thermo Trace 1310-TSQ 8000 gas chromatograph system coupled with mass spectrometer. Each 1 μL of derivatized sample was injected and separated with a TG-5MS GC column (0.25 mm × 30 m × 0.25 μm, Thermo Fisher, San Jose, CA, USA) in split mode with a 20:1 ratio. Helium was used as the carrier gas and was maintained at a constant flow of 1.2 mL/min. The GC temperature method is as follows: 60 °C for 1 min; increased to 320 °C at 20 °C/min; held at this temperature for 5 min. The electron energy was 70 eV and MS data were acquired in full-scan mode with a mass range of 50–500 m/z and a time range of 3.5–19 min.

## 5. Data Processing and Statistical Analysis

GC-MS data (raw data files) of all samples were converted to ABF format by using ABF Converter (http://www.reifycs.com/AbfConverter/) [32] and all these data were imported into MS-DIAL (v.2.7.2) software program [33] for peak detection, identification and alignment. Data was normalized by sum and transformed by log with Metaboanalyst (http://www.metaboanalyst.ca/) before a series of multivariate statistical analysis.

Statistical significance of the identified metabolites was assessed using one-way ANOVA test. Therefore, *p* ˂ 0.05 was set as the level of statistical significance. The metabolites were considered as potential biomarkers according to the threshold of Fold Change (FC) > 1.5 as well as *p* < 0.05. All statistical analysis was performed by using Prism 7 (GraphPad 7.00) and Metaboanalyst 4.0.

## 6. Conclusions

In this study, we first investigated the antipyretic and anti-inflammation effect of JXOL by measuring rectal temperature and inflammatory cytokines, respectively. Afterwards, we performed research on the brain metabolic profiling and biomarkers of yeast-induced fever in young rats. Finally, we selected 22 metabolites as potential biomarkers of yeast-induced fever and 12 metabolites as biomarkers of antipyretic mechanism of JOXL. In JXG, the imbalanced production of 3-phosphoglycerate, 5′-deoxy-5′-methylthioadenosine, adenine, beta-hydroxybutyric acid, glyceric acid, inosine, *N*-acetyl-d-galactosamine, *N*-acetyl-l-aspartic acid, pantothenic acid, pyruvic acid, quinolinic acid, tagatose, and uracil were regulated to normal, which can elucidate the mechanisms of antipyretic of JXOL to some extent.

In summary, this work was of significant importance in gaining insight into the brain metabolomics of pyretic rats and exploring the metabolic mechanism of the antipyretic effect of JXOL, which also provides a new strategy for further understanding the therapeutic mechanism of TCM.

## Figures and Tables

**Table 1:** Potential biomarkers of pyrexia.

NO.	Metabolites	HMDB	KEGG	FC NG vs. MG	*p* Value:		
					MG vs. NG	IBG vs. MG	JXG vs. MG
1	3-phosphoglycerate	HMDB0000807	C00597	0.455	<0.0001 ^##^	0.0040 **	<0.0001 **
2	5-6-dihydrouracil	HMDB0000076	C00429	1.506	0.0010 ^##^	<0.0001 **	0.8870
3	5′-deoxy-5′-methylthioadenosine	HMDB0001173	C00170	1.705	0.0020 ^##^	<0.0001 **	<0.0001 **
4	adenine	HMDB0000034	C00147	1.814	0.0080 ^##^	0.0400 *	<0.0001 **
5	adenosine	HMDB0000050	C00212	0.658	<0.0001 ^##^	1.0000	0.1070
6	adenosine-5-monophosphate	HMDB0000045	C00020	1.734	<0.0001 ^##^	0.1380	0.6430
7	alpha-aminoadipic acid	HMDB0000510	C00956	0.623	<0.0001 ^##^	0.8180	0.8340
8	arabitol	HMDB0000568	C01904	1.939	0.0470 ^#^	0.0470 *	0.4550
9	aspartate	HMDB0000191	C00049	0.589	<0.0001 ^##^	0.0400 *	0.1390
10	beta-hydroxybutyric acid	HMDB0000357	C01089	0.631	0.0080 ^##^	0.5000	0.0010 **
11	creatine	HMDB0000064	C00300	1.925	<0.0001 ^##^	<0.0001 **	0.8870
12	fructose-1-6-bisphosphate	HMDB0001058	C00354	2.419	<0.0001 ^##^	0.0020 **	0.5190
13	glyceric acid	HMDB0000139	C00258	0.582	<0.0001 ^##^	<0.0001 **	<0.0001 **
14	inosine	HMDB0000195	C00294	2.273	<0.0001 ^##^	<0.0001 **	<0.0001 **
15	*N*-acetyl-d-galactosamine	HMDB0000212	C01074	0.566	<0.0001 ^##^	<0.0001 **	<0.0001 **
16	*N*-acetyl-l-aspartic acid	HMDB0000812	C01042	1.541	<0.0001 ^##^	<0.0001 **	<0.0001 **
17	pantothenic acid	HMDB0000210	C00864	0.673	<0.0001 ^##^	0.0020 **	<0.0001 **
18	phosphogluconic acid	HMDB0001316	C00345	1.624	<0.0001 ^##^	0.0010 **	0.4300
19	pyruvic acid	HMDB0000243	C00022	0.576	<0.0001 ^##^	<0.0001 **	<0.0001 **
20	quinolinic acid	HMDB0000232	C03722	2.116	<0.0001 ^##^	0.0010 **	<0.0001 **
21	tagatose	HMDB0003418	C00795	0.391	<0.0001 ^##^	0.0030 **	<0.0001 **
22	uracil	HMDB0000300	C00106	0.588	<0.0001 ^##^	0.0020 **	<0.0001 **

# *p* < 0.05, ## *p* < 0.01, vs. NG; * *p* < 0.05, ** *p* < 0.01, vs. MG.
